# Epidemiology and Long-Term Outcomes in Thoracic Transplantation

**DOI:** 10.3390/jcdd10090397

**Published:** 2023-09-18

**Authors:** Abey S. Abraham, Manila Singh, Matthew S. Abraham, Sanchit Ahuja

**Affiliations:** 1 Department of Cardiothoracic Anesthesiology, Cleveland Clinic Foundation, Cleveland, OH 44195, USA; abey_27@hotmail.co.uk (A.S.A.); singhm8@ccf.org (M.S.); 2 School of Medicine, University of Leeds, Leeds LS2 9JT, UK; abra3520@gmail.com

**Keywords:** lung disease, lung transplantation, lung transplant survival, chronic lung allograft dysfunction, primary graft dysfunction

## Abstract

Over the past five decades, outcomes for lung transplantation have significantly improved in the early post-operative period, such that lung transplant is now the gold standard treatment for end-stage respiratory disease. The major limitation that impacts lung transplant survival rates is the development of chronic lung allograft dysfunction (CLAD). CLAD affects around 50% of lung transplant recipients within five years of transplantation. We must also consider other factors impacting the survival rate such as the surgical technique (single versus double lung transplant), along with donor and recipient characteristics. The future is promising, with more research looking into ex vivo lung perfusion (EVLP) and bioengineered lungs, with the hope of increasing the donor pool and decreasing the risk of graft rejection.

## 1. Introduction

Trailblazing into the realm of medical miracles, James Hardy etched his name in history in 1963 by successfully demonstrating that a lung transplant was surgically feasible [1]. Even though the initial patient could only grasp three additional weeks of life before succumbing to complications, this pivotal moment marked the beginning of an incredible journey toward medical advancement. The following decades, riddled with false starts and heartbreaking failures, were a testament to the resilience of the medical community, persisting despite setbacks that were predominantly due to anastomotic failure.

From this bedrock of trials, the first glimmers of success emerged with single lung transplants (SLTs), swiftly culminating in a double lung transplant (DLT) in 1986 [1]. Since then, the frequency and success rates of lung transplants have been on an uphill trajectory globally, thanks to leaps in technology and practice. The advent of powerful immunosuppressive agents, notably cyclosporine, coupled with enhanced patient management techniques, dramatically improved the prognosis post-transplant.

Fast forward to 2017, and the landscape has transformed significantly. As per the International Society for Heart and Lung Transplantation (ISHLT), over 4500 lung transplants were performed annually worldwide—a testament to advanced organ allocation protocols. Nevertheless, the demand for organs still towers above the supply, pointing to a persistent and pressing need for solutions in organ transplantation. The introduction of newer techniques like EVLP has expanded the organ pool. This review focuses mainly on discussing EVLP and bioengineering, along with outcomes of lung transplantation.

## 2. Single Versus Double Lung Transplant

When looking through the literature, there are no randomized controlled trials specifically comparing single versus double lung transplants for ethical reasons. In addition, there are the technical aspects of the procedure and the consideration of the recipients’ underlying lung disease. Taking a step back, knowing which disease entities warrant single versus double lung transplantation is important. Double lung transplant indications include cystic fibrosis and bronchiectasis; with these conditions, a single lung transplant is an absolute contraindication given the risk of contamination of the new lung. For most other diseases, such as interstitial lung disease, emphysema, and primary or secondary pulmonary hypertension, it appears that single versus double lung transplantation comes down to institute preference [2]. 

Looking at the ISHLT 2017 report, they analyzed 53,396 lung transplants over 15 years; they concluded that double lung transplantation revealed a higher one-year, three-year, five-year and ten-year survival rate. Furthermore the difference in the rate seemed to increase over the ten-year period. In this study, the main indication for transplantation was either chronic obstructive pulmonary disease (COPD) or ILD [3]. Schaffer et al. conducted a comparative study in 2015, analyzing around 7000 patients over 7 years [4]. They looked at outcomes for single vs. double lung transplantation for idiopathic pulmonary fibrosis (IPF) and COPD. They found that double lung transplantation was associated with better graft survival in the IPF group. The team found no significant difference between single versus double transplantation in the COPD group for graft survival at 5 years. In 2001, Meyer et al. performed a comparative study looking at 2260 patients over 6 years, mainly single versus bilateral lung transplants for COPD patients. They concluded that bilateral sequential lung transplants demonstrated a higher survival rate for individuals under 60 years of age [5]. 

We must also look into the development of bronchiolitis obliterans syndrome (BOS), one of the two phenotypes of CLAD, the other being restrictive allograft syndrome (RAS). Hadjialiadis et al. performed a single-center study; even by controlling the baseline characteristics between the two groups, they found that the incidence of BOS was higher with single lung transplantation [6]. Neurohr et al. conducted a study looking at lung transplantation for pulmonary fibrosis; they reported that SLTs were associated with an increased risk of BOS [6]. On the other hand, other studies, such as Meyer et al., found no difference in the development of BOS between SLT and DLT groups [5]. Unlike the initial two single-center studies, the Meyer et al. study had a larger sample size of 2260 lung transplant recipients. 

As seen above, the results in the literature could be more consistent concerning long-term survival and BOS development. Many authors performed comparative studies using data from the comprehensive ISHLT database. Given the unique nature of lung transplantation, there appears to be far too many variables to control for when considering donor and recipient characteristics. 

## 3. Ex Vivo Lung Perfusion (EVLP) 

EVLP has established itself as a formidable intervention in helping to reduce the demand–supply mismatch of viable lungs for transplantation. Jirsch et al. first proposed and tested EVLP in 1970 through animal testing, and over the past 50 years, significant advancements have been made, including its successful use in human transplantation in 2001 [7,8]. This revolutionary change has allowed for timely evaluations following explant, and has revitalized previously discarded donor lungs. 

**Figure 1:**
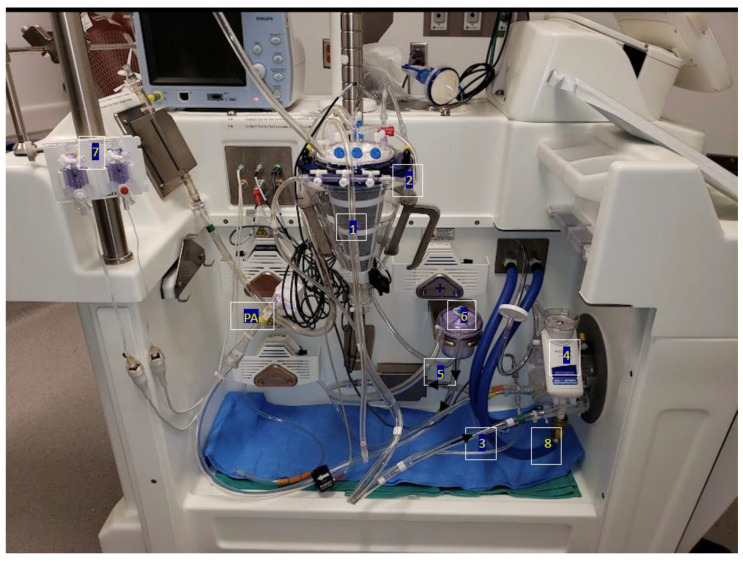
EVLP circuit. 1 = reservoir, 2 = sampling port, 3 = inflow to oxygenator, 4 = oxygenator, 5 = outflow (oxygenator to leukocyte filter), 6 = leukocyte filter, 7 = pressure lines for left atrium (LA) and pulmonary artery (PA), 8 = water lines.

**Figure 2:**
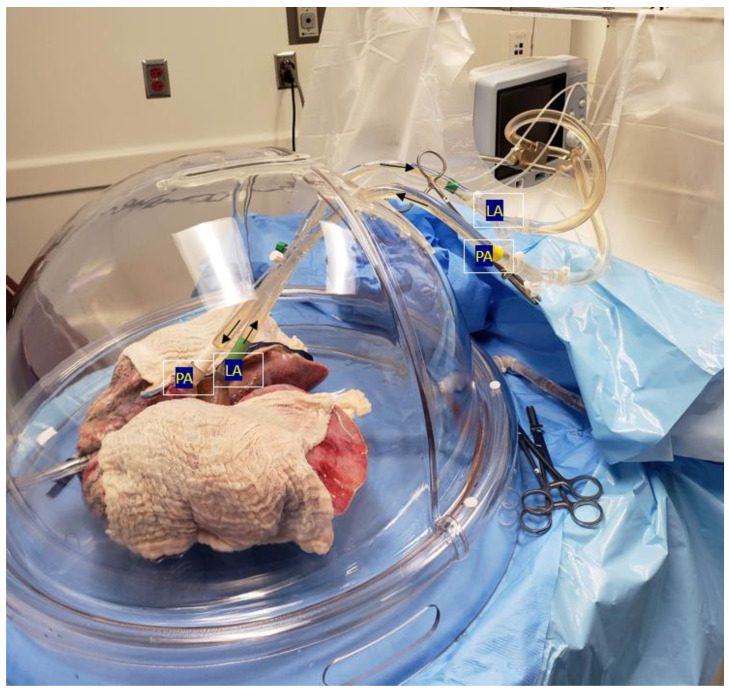
Donor lungs with labelled inflow to the pulmonary artery (PA) and outflow from the left atrium (LA).

EVLP is practiced across three different protocols. These include Lund, Toronto, and the OCS (Organ Care System) [9]. Whilst Lund originated EVLP in clinical practice, it has been further refined into the Toronto Technique, which is most commonly used today. The procedure starts with an initial circuit containing 2 L of perfusate solution, 500 mg of methylprednisolone, and 500 mg of imipenem and cilastatin. Upon the retrieval of the donor’s lungs, left atrial access is made by suturing a cannula to the cuff with prolene sutures. Following this, pulmonary artery access is established by inserting a cannula just before the artery bifurcation, held with silk ties. Where the donor artery is not retrieved, a conical cannula can be inserted and held with prolene sutures. To prevent the deflation of the lungs, the trachea is clamped, and an endotracheal tube is inserted. Before attaching to the main circuit, a retrograde flush is passed through the venous line to flush out microthrombi and debris. Finally, the lungs are placed within the dome, and the circuit is attached. Before running the perfusate, air must be removed from the lungs. After this, the cannulas are attached to the main circuit, and incrementally, flow is established, aiming for a 40% flow rate of the donor cardiac output. Simultaneously, normothermia is achieved at 37 degrees Celsius. Finally, once the circuit is optimized at physiological parameters, the clamp is removed, and the lungs can ventilate. The circuit allows for continuous arterial blood gas (ABG) and venous blood gas (VBG) monitoring, including the pre- and post-oxygenation values. Additionally, bronchoscopy and X-ray imaging are used to identify growing lesions. Please refer to Figure 1 and Figure 2 outlining the EVLP circuit. 

Whilst EVLP’s largest benefit has been to increase donor organ supply through the desirable restitution of lung physiology the post-operative outcomes are still yet to be thoroughly explored. The main hurdle thus far is the rate-limiting factor of prolonged ischemia time due to the transport of the lungs from the donor to the recipient.

Cypel et al. published a review looking at the lung transplantation of high-risk donor lungs with the use of EVLP [10]. They deemed the term ‘high risk’ using a five-point criteria, which included a PaO_2_:FiO_2_ ratio of less than 300 mmHg, the presence of pulmonary edema, and donation after circulatory death (DCD). Their primary outcome was the development of primary graft dysfunction within 72 h post-operatively. There were numerous secondary outcomes, such as duration of hospital and critical care stay, 30-day mortality, and mechanical ventilation duration. The study included 20 lungs that underwent EVLP compared with 116 lungs that were procured in a conventional manner. The team found no significant difference in either group for the primary and secondary outcomes. 

Divithotawela et al. (2019) conducted a nine-year cohort study assessing the post-operative outcomes of 230 EVLP versus 706 control thoracic transplant patients [11]. It was observed that EVLP donor lungs had higher rates of injury, with significantly lower PaO_2_ values, higher incidences of abnormal CXRs, and higher proportions of significant smoking histories. Despite this, there were no significant differences in the time to chronic lung allograft dysfunction (CLAD) or the survival time of the allograft itself. The study concluded that EVLP increases the donor pool with negligible differences in post-operative outcomes. Additionally, Tian et al. analyzed eight studies observing the outcomes of 1191 patients between EVLP and non-EVLP lung transplants [12]. The metanalysis fortified the aforementioned results, demonstrating similar outcomes between both methods, except for poorer donor lungs noted in EVLP cases. These included poorer PaO_2_/FiO_2_ values and higher smoking rates in donors. Outcome measures included the length of time on ECMO (extracorporeal membrane oxygenation), the length of time in intensive care, and graft disorder 72 h post-transplant. While physiological metrics and pathological markers are the main vehicles of assessment, Tikkanen et al. also evaluated the quality of life post-transplant [13]. In their study, the specific criteria included the difference in meters walked over six minutes, and the maximum predicted FEV1 values were utilized. Both measures observed no significant differences in either metric across 340 conventional and 63 EVLP transplants. 

Notably, other primary research suggests that the effects of EVLP are potentially dependent on the cumulative caseload of transplant centers. Chen et al. (2023) analyzed the data set of 9708 normal transplants against 553 EVLP transplants, stratified into high versus low-case EVLP centers [14]. It was observed that EVLP centers with lower caseloads (<15 transplants over the four-year study period) were more likely to have comparably poorer outcomes in the percentages of 1-year survival compared to their conventional transplant counterparts. This difference was not observed in larger centers. Only 6% of transplants are EVLP-based in the United States, according to the United Network for Organ Sharing (UNOS). As this system falls in the minority of practice, adequate and routine training is crucial for optimal graft management. If operations are few and far between, and exacerbated at centers with low existing caseloads, this is possibly attributed to poorer outcomes, especially since the same study noted no significant differences in outcomes between the conventional thoracic transplants. 

Despite this, the outlook of EVLP is generally favorable, which is partly owed to EVLP’s ability to correct pathologies in procurement. Nakajima et al. explain this further in their 2021 review article; with the high risk of corrective surgery in vivo, EVLP offers the vital opportunity to operate without the added risks, as well as to isolate the lungs without risk to other organs [15]. As function is assessed and operative correction is made, it allows for predictable outcomes. Furthermore, the review exemplifies this through evidenced and treated pathologies, such as using alteplase in donor pulmonary emboli. The fibrinolytic effect was enhanced as there was no additional risk of bleeding compared to being treated in vivo. Additionally, through imaging and bronchoscopy, cases of pneumonia and pulmonary edema have been treated, too. Sanchez et al. (2014) exhibited this in a case report of successful reconditioning following neurogenic pulmonary edema [16]. Despite donor lungs being rejected from numerous centers due to a PaO_2_ value of 188 mmHg, once poor lung compliance and increased pulmonary vascular resistance had been corrected, improving oxygenation alongside reperfusion at normothermia reflected the correction of the pulmonary edema. The patient made a rapidly successful recovery, and was discharged 15 days later. The report demonstrates EVLP’s use in expanding the donor pool, as well as ex vivo perfusion in correcting pathology and its comparative outcomes to normal transplantation. The risk of recipient infection from donor disease is possible in all transplant cases [17]. The magnitude of this risk increases with poor ciliary clearance and extended times on mechanical ventilation. It is further exacerbated when patients are multimorbid, and medication damages other systems, especially for the possibility of multiple organs being donated, too. Part of the myriad benefits that EVLP brings in addressing such issues includes its allowance for increased drug volumes and concentrations without increasing the risk of morbidity to the recipient or donor. An example given by Ahmad et al. (2022) includes the administration of vancomycin [18]. The therapeutic range is given at 10 mg/L; however, any increases past 30 mg/L risk acute renal failure. However, for a 75 kg male donor, whose lungs are placed in EVLP, a 1125 mg dose can be administered (15 mg/kg) to allow for a constant concentration of 225 mg/L running through the circuit, remarkably higher than the concentration dose for nephrotoxicity, which provides maximum antibiotic therapy in the light of perfusing lungs ex vivo. These results are evidenced in prior research by Andreasson et al., who looked into the effect of EVLP on microbial load in 18 lung donors [19]. Microbial samples were collected using a bronchoalveolar lavage with 40 mL NaCl before aspirate samples were taken, and the lungs were allowed to ventilate and perfuse via EVLP. Thirteen donor lungs cultured microbial growth, both anaerobic and aerobic, as well as six donor lungs culturing yeast strains. The EVLP perfusate included amphotericin B and meropenem (to which all fungal species and bacteria were sensitive, respectively). The microbial and fungal loads significantly decreased upon sampling post-perfusate after treatment was given. 

In conclusion, EVLP has certainly helped to boost the donor pool; furthermore, it has not been associated with poorer outcomes compared to conventional transplantation, based on our literature review. Moreover, the use of EVLP has allowed teams to successfully treat conditions such as pneumonia and pulmonary edema in donors’ lungs, thus enabling them to be transplanting viable organs. 

## 4. Bioengineered Lungs and Organ Repair Centers: Expanding Possibilities for Transplantation

We mentioned earlier that the introduction of EVLP has revolutionized the field of lung transplantation by facilitating the maintenance of harvested lungs in a viable state. This technique has considerably expanded the pool of organs available for transplantation, overcoming the limitations of conventional cold organ preservation methods. In contrast to cold preservation, EVLP preserves lungs at normothermic conditions, thereby enabling the opportunity for pre-implantation organ repair and regeneration, which we refer to as bioengineering. This innovative approach holds great promise in enhancing organ survival rates, reducing rejection occurrences, and facilitating more flexible transplant scheduling.

The underlying principle of lung bioengineering involves decellularizing retrieved lungs, and a subsequent recellularization using either autologous or allogeneic mesenchymal stem cells, utilizing the lung scaffold as a platform. This process is particularly significant in expanding the donor pool, which is currently restricted due to a high incidence of lung injury, especially among trauma patients. Targeted regenerative therapies applied to these injured lungs can potentially increase the availability of viable organs for transplantation. 

There is growing research on the various lung tissue scaffolds, either biological (acellular) or artificial (synthetic), and each has its benefits and limitations. First, we must understand the purpose of the scaffold; it essentially functions as the extracellular matrix (ECM), and it provides structural integrity to the tissue as well as the template for the recellularization process. The main benefit associated with biological scaffolds is the preservation of the complex architecture of the lung, as well as the retention of the ECM. We do not know exactly the extent to how much this ECM is affected with the decellularization process; however, there is relatively more preservation when compared to artificial scaffolds. The biological scaffold technique carries the risk of infection; furthermore, its use is limited by the shortage of donor lungs. To combat this, in the future, we may have to use xenogeneic (animal) ECM as the scaffold [20]. There has been research looking into use of porcine lungs; however, there are concerns of α-galactosyl epitope in the ECM potentially eliciting a rejection response. Furthermore, more research needs to be conducted into the surgical technique, especially with respect to the anastomosis of the bronchi and pulmonary blood vessels [21]. 

There are numerous techniques to manufacture an artificial scaffold, such as 3D bioprinting, bioreactors, and electrospinning. The benefits associated with artificial scaffolding are the ability to design and create a specific scaffold, along with not having to rely on the donor organ pool. In the past decade, 3D bioprinting has made advances; it is a technique that relies on using laser- and ink-jet-based technology to create a scaffold. So far, there has been some success in bioprinting the trachea as per some animal studies. However, no trials have looked into bioprinting lung tissue [22]. In theory, this technique should allow one to create as close of a replica of native lung tissue, however at present, this warrants further advances in technology. Of note, the specific limitations include an inability to successfully create a gas exchange interface, along with the creation of lung vasculature and alveolar epithelium. Furthermore, modern day bio-ink does not truly function like an ECM; hence, research is being conducted looking into possible hybrid solutions. Bioreactors are devices that provide the ideal conditions for growth or reactions to occur; in this setting, the bioreactor will allow for decellularization, recellularization, and lung maturation. Limitations of this technique include optimizing factors pertaining to homeostasis, such as ideal temperature management within the device, along with controlling the pH, ventilation, and perfusion. Finally, electrospinning is a modality whereby nanoscale fibers are created; these act as a scaffold for cell adhesion and attachment. The hope with electrospinning is that, by controlling the nanofiber dimensions, one can create the ideal extracellular matrix. This modality has thus far only been used in vitro, mainly to produce trachea scaffolds, with no published reports on its use in bioengineered lungs [20,23]. The main limitations include mechanical strength along with concerns of the toxicity of the nanofiber scaffold. 

In 2015, Tan et al. demonstrated the successful revascularization and re-epithelialization of an implanted lung tissue scaffold [24]. This led to the prolonged survival of a patient with a very limited life prognosis, who eventually succumbed to cancer recurrence after over a year. While this human case study demonstrates the potential of the technique, further advancements are necessary to ensure its feasibility and readiness for human trials.

Bioengineering offers promising avenues for enhancing transplanted organs through genetic therapy, regenerative stem cell therapy, and pharmacotherapy to address infections. By leveraging these approaches, the functionality and viability of transplanted organs can be improved, thereby enhancing patient outcomes and organ availability.

Building upon the pioneering work of Toronto General Hospital in the field of EVLP, the concept of Organ Repair Centers has emerged as a novel approach to facilitate the bioengineering of organs prior to transplantation. Currently, centers located in Silver Spring, Maryland, as well as Jacksonville, Florida, serve as central hubs catering to large geographic regions, providing the essential infrastructure required for this process. The fundamental concept entails transporting harvested organs to these specialized centers, where they undergo bioengineering procedures before being prepared for implantation. Although the costs associated with these additional maneuvers and tools, such as bioreactors and 3D printers for scaffold printing, are substantial, the potential benefits offered by organ repair centers are extensive and far-reaching. Establishing these centers opens up new possibilities for advancing the field of organ transplantation.

## 5. Conclusions 

With an increasing number of individuals with end-stage lung failure, aside from lung transplant being curative, we must find ways to either boost the organ donation pool or ration the available resources. We still do not have unanimous data to support the use of single versus double lung transplants with regard to long-term survival. The reality is there are multiple factors at play, and it is unlikely we will see a direct, randomized, and controlled trial comparing the two techniques. EVLP demonstrates a notable propensity to augment the supply of viable lung donors, and execute comparable outcomes to conventional transplant techniques. We have observed this in comparable incidences of CLAD and allograft dysfunction post-transplant. Whilst still in its infancy, it serves as a beacon of transformation in lung transplantation through the assessment and restitution of marginal donors, through the correction of pulmonary edema, diagnosed infection, and other noted pathologies. Further research is needed to determine the effectiveness of sub-techniques within EVLP, such as portable machines versus center equipment, compared to external centers. This becomes especially pertinent when transport logistics is vital in organ procurement and explant, and centers are resource- and practice-dependent. Furthermore, we live in an age where technology is rapidly advancing. Maybe bioengineered lungs will become more prevalent in the future; only time will tell. The use of 3D bioprinting and electrospinning to create customized lungs, along with xenogeneic potential for scaffolding, are all exciting areas of research in the coming years. 

## Data Availability

Not applicable.

## References

[1] Dabak G., Şenbaklavacı Ö. (2016). History of Lung Transplantation. Turk. Thorac. J..

[2] Puri V., Patterson G.A., Meyers B.F. (2015). Single versus bilateral lung transplantation: Do guidelines exist?. Thorac. Surg. Clin..

[3] Lund L.H., Khush K.K., Cherikh W.S., Goldfarb S., Kucheryavaya A.Y., Levvey B.J., Meiser B., Rossano J.W., Chambers D.C., Yusen R.D. (2017). The Registry of the International Society for Heart and Lung Transplantation: Thirty-fourth Adult Heart Transplantation Report-2017; Focus Theme: Allograft ischemic time. J. Heart Lung Transplant..

[4] Schaffer J.M., Singh S.K., Reitz B.A., Zamanian R.T., Mallidi H.R. (2015). Single- vs double-lung transplantation in patients with chronic obstructive pulmonary disease and idiopathic pulmonary fibrosis since the implementation of lung allocation based on medical need. JAMA.

[5] Meyer D.M., Bennett L.E., Novick R.J., Hosenpud J.D. (2001). Single vs bilateral, sequential lung transplantation for end-stage emphysema: Influence of recipient age on survival and secondary end-points. J. Heart Lung Transplant..

[6] Hadjiliadis D., Davis R.D., Palmer S.M. (2002). Is transplant operation important in determining posttransplant risk of bronchiolitis obliterans syndrome in lung transplant recipients?. Chest.

[7] Jirsch D.W., Fisk R.L., Couves C.M. (1970). Ex vivo evaluation of stored lungs. Ann. Thorac. Surg..

[8] Steen S., Sjöberg T., Pierre L., Liao Q., Eriksson L., Algotsson L. (2001). Transplantation of lungs from a non-heart-beating donor. Lancet.

[9] Possoz J., Neyrinck A., Van Raemdonck D. (2019). Ex vivo lung perfusion prior to transplantation: An overview of current clinical practice worldwide. J. Thorac. Dis..

[10] Cypel M., Yeung J.C., Liu M., Anraku M., Chen F., Karolak W., Sato M., Laratta J., Azad S., Madonik M. (2011). Normothermic ex vivo lung perfusion in clinical lung transplantation. N. Engl. J. Med..

[11] Divithotawela C., Cypel M., Martinu T., Singer L.G., Binnie M., Chow C.W., Chaparro C., Waddell T.K., de Perrot M., Pierre A. (2019). Long-term Outcomes of Lung Transplant with Ex Vivo Lung Perfusion. JAMA Surg..

[12] Tian D., Wang Y., Shiiya H., Sun C.B., Uemura Y., Sato M., Nakajima J. (2020). Outcomes of marginal donors for lung transplantation after ex vivo lung perfusion: A systematic review and meta-analysis. J. Thorac. Cardiovasc. Surg..

[13] Tikkanen J.M., Cypel M., Machuca T.N., Azad S., Binnie M., Chow C.W., Chaparro C., Hutcheon M., Yasufuku K., de Perrot M. (2015). Functional outcomes and quality of life after normothermic ex vivo lung perfusion lung transplantation. J. Heart Lung Transplant..

[14] Chen Q., Malas J., Krishnan A., Thomas J., Megna D., Egorova N., Chikwe J., Bowdish M.E., Catarino P. Limited cumulative experience with ex vivo lung perfusion is associated with inferior outcomes after lung transplantation. J. Thorac. Cardiovasc. Surg..

[15] Nakajima D., Date H. (2021). Ex vivo lung perfusion in lung transplantation. Gen. Thorac. Cardiovasc. Surg..

[16] Sanchez P.G., Iacono A.T., Rajagopal K., Griffith B.P. (2014). Successful lung salvage by ex vivo reconditioning of neurogenic pulmonary edema: Case report. Transplant. Proc..

[17] Bunsow E., Los-Arcos I., Martin-Gómez M.T., Bello I., Pont T., Berastegui C., Ferrer R., Nuvials X., Deu M., Peghin M. (2020). Donor-derived bacterial infections in lung transplant recipients in the era of multidrug resistance. J. Infect..

[18] Ahmad K., Pluhacek J.L., Brown A.W. (2022). Ex Vivo Lung Perfusion: A Review of Current and Future Application in Lung Transplantation. Pulm. Ther..

[19] Andreasson A., Karamanou D.M., Perry J.D., Perry A., Ӧzalp F., Butt T., Morley K.E., Walden H.R., Clark S.C., Prabhu M. (2014). The effect of ex vivo lung perfusion on microbial load in human donor lungs. J. Heart Lung Transplant..

[20] Shakir S., Hackett T.L., Mostaço-Guidolin L.B. (2022). Bioengineering lungs: An overview of current methods, requirements, and challenges for constructing scaffolds. Front. Bioeng. Biotechnol..

[21] Tsuchiya T., Sivarapatna A., Rocco K., Nanashima A., Nagayasu T., Niklason L.E. (2014). Future prospects for tissue engineered lung transplantation: Decellularization and recellularization-based whole lung regeneration. Organogenesis.

[22] Frejo L., Grande D.A. (2019). 3D-bioprinted tracheal reconstruction: An overview. Bioelectron. Med..

[23] Lemon G., Lim M.L., Ajalloueian F., Macchiarini P. (2014). The development of the bioartificial lung. Br. Med. Bull..

[24] Tan Q., Liu R., Chen X., Wu J., Pan Y., Lu S., Weder W., Luo Q. (2017). Clinic application of tissue engineered bronchus for lung cancer treatment. J. Thorac. Dis..

