# Correction: Abraham et al. Epidemiology and Long-Term Outcomes in Thoracic Transplantation. *Journal of Cardiovascular Development and Disease* 2023, *10*, 397

**DOI:** 10.3390/jcdd10120493

**Published:** 2023-12-12

**Authors:** Abey S. Abraham, Manila Singh, Matthew S. Abraham, Sanchit Ahuja

**Affiliations:** 1 Department of Cardiothoracic Anesthesiology, Cleveland Clinic Foundation, Cleveland, OH 44195, USA; abey_27@hotmail.co.uk (A.S.A.); singhm8@ccf.org (M.S.); 2 School of Medicine, University of Leeds, Leeds LS2 9JT, UK; abra3520@gmail.com; 3 Department of Cardiothoracic Anesthesiology, Outcomes Research Consortium, Cleveland Clinic, Cleveland, OH 44195, USA

In the published work [1], there was an error regarding the affiliation for “Sanchit Ahuja”. The updated affiliation should read as follows:

Affiliation 3: Department of Cardiothoracic Anesthesiology, Outcomes Research Consortium, Cleveland Clinic, Cleveland, OH 44195, USA.

The authors state that the scientific conclusions are unaffected. This correction was approved by the Academic Editor. The original publication has also been updated.
